# Impact of case-specific learning goal on robotic-assisted surgery care delivery

**DOI:** 10.1007/s44186-026-00514-6

**Published:** 2026-03-19

**Authors:** Michael Meara, Theresa Wang, Heidi Pieper, David Renton, Xiaodong Phoenix Chen

**Affiliations:** Department of Surgery, The Ohio State University, Columbus, OH 395 W 12th Ave43210 USA

**Keywords:** Surgical education, Resident training, Robotic-assisted surgery, Learning goal

## Abstract

**Introduction:**

We hypothesize that resident’s intraoperative case-specific learning goal may influence resident intraoperative autonomy and robotic-assisted surgery (RAS) surgical outcomes measured by operative time (OT), length of stay (LOS), direct cost (DC), and 30-day readmission (30R).

**Methods:**

Valid resident operative performance evaluations, which includes case-specific learning goal selection (LGS) and degree of learning goal completion (LGC) metrics, of three outpatient RAS procedures—inguinal hernia, ventral hernia, and cholecystectomy—performed by PGY2–5 residents were collected. Cases in which residents served as bedside assistants were excluded. OT, LOS, DC, and 30R for matched cases were extracted from hospital records. Descriptive statistical analysis was applied.

**Results:**

A total of 104 evaluations from 57 outpatient RAS cases were analyzed. Residents’ overall permitted intraoperative autonomy was positively correlated with their LGS (0.66, *p* < 0.0001) and LGC (0.64, *p* < 0.0001). Overall LGS had minimal impact on OT, LOS, and DC. However, significant differences (all *p* < 0.05) in surgical outcomes were observed between different LGC scores: Compared to cases with an LGC score 5 (fully completed learning goal), cases with an LGC score 3 (partially completed learning goal) showed significantly longer LOS (8.00 h > 5.87 h) and OT (98.36 min > 74.49 min) as well as higher DC ($4471.84 > $3945.23). RAS cases with an LGC score 5 had a higher probability of 30R than those with an LGC score 3 (18.03% > 1.64%), though it was not significant. LGS also showed a similar trend.

**Conclusions:**

Study findings suggest the case-specific learning goal may influence resident autonomy as well as LOS, OT, DC and 30R of outpatient RAS cases. Identifying an achievable learning goal upon a resident’s competency level may enhance intraoperative training and RAS care outcomes.

## Introduction

The use of robotic-assisted surgery (RAS) in surgical residency training is rapidly increasing [1]. Robotic inguinal hernia (RIH), robotic cholecystectomy (RC), and robotic ventral hernia (RVH) are three RAS procedures that are gradually being used to train a general surgery resident in academic medical centers and teaching hospitals. RAS—especially RAS with a dual console—provides convenience (e.g., pass control back and forward) for attending surgeons to facilitate teaching and grant resident autonomy in the operating room (OR) [2]. But multiple studies reported that guidelines for RAS training and intraoperative teaching were still needed [3, 4].

However, intraoperative teaching in an RAS case is a complex task with a high cognitive demand [5] and many distractors, potentially leading to detrimental clinical outcomes, such as prolonged surgery time length, increased 30-day complication rates and composite morbidity [6–9]. Although intraoperative teaching does not significantly compromise major patient safety, several factors (e.g., resident training level) may contribute to detrimental surgical outcomes based on literature [10]. But which intraoperative teaching element(s) can be improved to enhance RAS surgical outcomes remains unclear.

Traditionally, the case-specific learning goal has been a key element of intraoperative teaching in the OR [11]. A learning goal is typically the same as a resident’s primary learning or practice task in a surgical case. A clearly defined, achievable learning goal may improve residents’ intraoperative learning experience and autonomy [12]. Therefore, we hypothesize that a case-specific learning goal will not only influence resident intraoperative autonomy but also impact RAS surgical outcomes measured by operative time (OT), length of stay (LOS), direct cost (DC) [13], and 30-day readmission (30R). The goal of this preliminary study is to test this hypothesis. Study findings could potentially contribute to enhancing RAS surgical outcomes without sacrificing resident intraoperative training.

## Methods

### Setting and participants

This study was conducted in the Center for Minimally Invasive Surgery at an academic medical center. General surgery residents from postgraduate year (PGY) 2 to 5 and RAS faculty from Department of Surgery were eligible to participate in this study. All general surgery residents in our institution must complete a robotic surgery training curriculum prior to being permitted to operate as a console surgeon. The robotic training curriculum consists of three parts per the Intuitive training requirements for residents and fellows [14]: two online training modules, one simulation training module (on the Da Vinci SimNow and score at least 90%), and the case logs of five RAS cases as bedside-assistant. The Institutional Review Board (IRB) approved this study (#2021H0154).

### Data collection and analysis

We collected resident operative performance evaluations with an augmented procedure-specific Surgical Entrustable Professional Activities (SEPA) instrument [15, 16], which contains a set of key evaluation metrics with a 1-to-5 Likert scale: case difficulty, the resident’s case-specific learning goal selection, the degree of learning goal completion, surgical timeout quality, procedure-specific skills (6 items), procedure step-specific guidance (6 items), general skills (4 items), overall guidance, operative planning and judgment, overall technical performance, overall team management, and prospective entrustment (i.e., future entrustment the attending surgeon would like to grant the resident in upcoming similar cases). Two case-specific learning goal metrics—learning goal selection (LGS) and degree of learning goal completion (LGC)—and two resident autonomy metrics from SEPA evaluations (Table 1) were included in current study.

**Table 1:** Descriptions of SEPA learning goal and resident autonomy metrics

SEPA evaluation items (Input Metrics)	Description and rating scale anchors
Case-specific learning goal (Learning Goal Selection Metric)	To assess whether the case-specific learning goal is manageable and appropriate for the resident and the case *Rating scale anchors:* 1—Had a global non-specific learning goal 3—Had a specific learning goal without manageable plans and/or tasks 5—Had a specific learning goal which was manageable and appropriate for this resident and the case
Achieved case specific learning goal (Learning Goal Completion Metric)	To assess whether the resident is able to complete the pre-identified learning goal by the end of the case *Rating scale anchors:* 1—Did not achieve learning goal 3—Completed some of the learning goal 5—Achieved 100% of the learning goal
Step-specific guidance (Step-Specific Autonomy Metric)	To assess the amount of guidance a resident needed from the attending surgeon in each procedure-specific critical step. Less guidance needed implies more autonomy *Rating scale anchors:* 1—Substantial guidance was provided/needed 3—Some guidance was provided/needed 5—Minimal guidance was provided/needed
Overall guidance (Overall Autonomy Metric)	To assess the overall amount of guidance provided to/received by this resident throughout the evaluated case *Rating scale anchors:* 1—Substantial guidance was provided/needed 3—Some guidance was provided/needed 5—Minimal guidance was provided/needed

A purposeful sampling approach was applied with following inclusion criteria: (1) RAS performed with a dual console Da Vinci® Xi™ surgical system (Intuitive Surgical Inc, Sunnyvale, CA), and (2) RIH, RVH, and RC cases only. Residents as bedside assistant cases were excluded. The operating resident and the attending surgeon were invited to complete the same SEPAs evaluation of resident performance in their eligible RAS case (RC, RIH, and RVH) within 3 days of each completed RAS operation via the online survey Qualtrics system (SAP SE, Provo, UT).

OT (i.e., cut-to-close time), DC, LOS (measured in hours), and 30R of matched RAS cases were retrospectively extracted from hospital records. Statistical analysis was performed using Pearson correlation, one-way ANOVA, and contingency table with JMP Pro (version 18; SAS Institute Inc, Cary, NC). Extreme sample comparison was applied to assess the impacts of the highest score and the lowest score on surgical outcomes. *P* < 0.05 is considered statistically significant. A retrospective power calculation (α = 0.05) was performed to determine the statistical power of study findings.

## Results

A total of 104 SEPA evaluations from 57 RAS cases (RIH: 28, RC: 23, and RVH: 6) were included. Most RAS cases (80.7%, 46 out of 57) involved a PGY5 resident, followed by PGY2 residents (12.3%, 7 out of 57). PGY4 residents participated in three RAS cases, and PGY3 residents participated in two RAS cases. The majority of included cases (82.5%, 47 out of 57) received evaluations from both attending surgeons and residents.

**Fig. 1:**
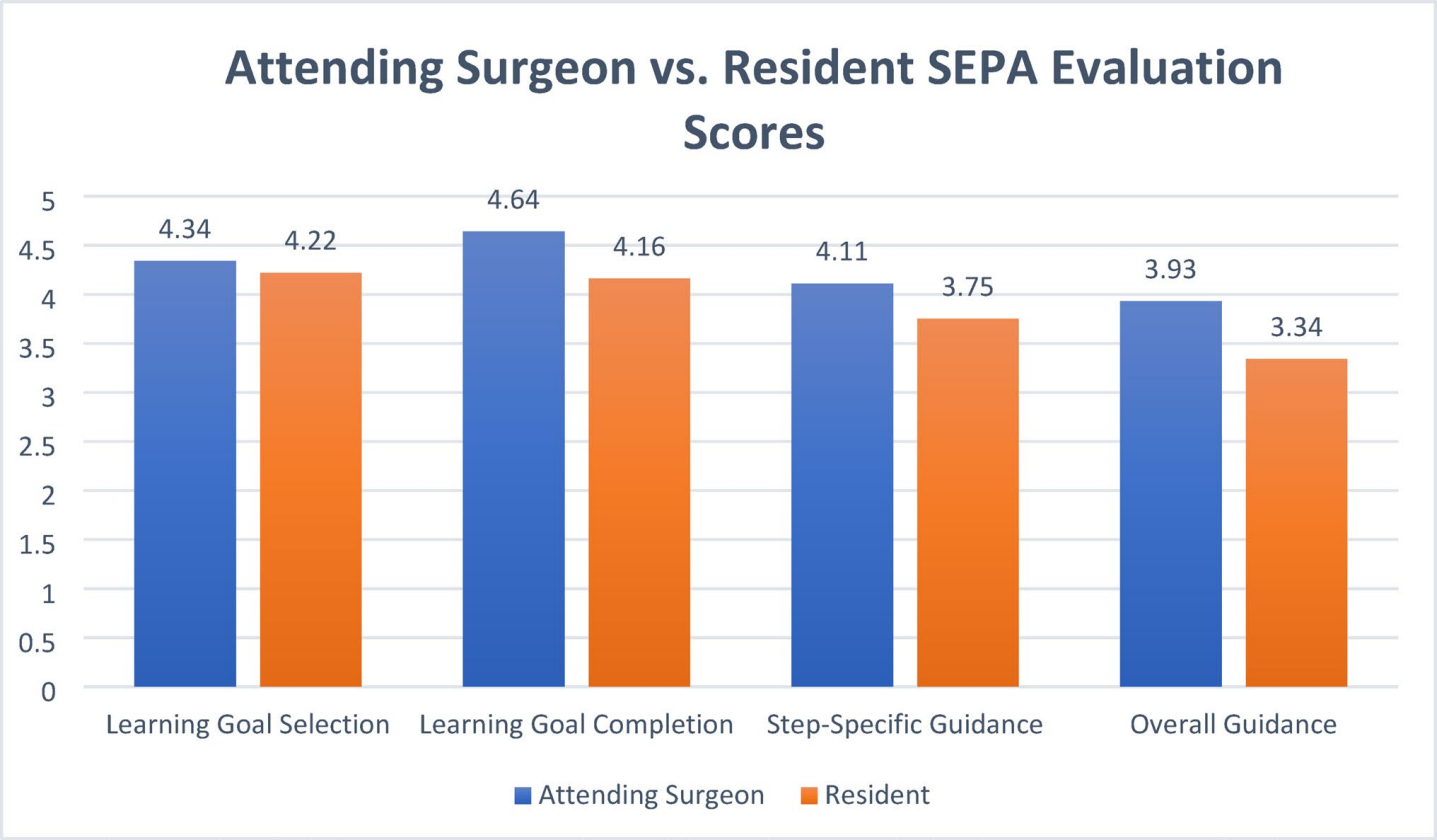
A comparison of attending surgeon and resident scores on four evaluative metrics

Residents and attending surgeons’ scores are similar with variations within the same Likert rating scale except for the “Step-Specific Guidance” metric (Fig. 1). Residents’ overall permitted intraoperative autonomy – scores of graded “Overall Guidance”—was positively correlated with their LGS (r = 0.66, *p* < 0.0001) and LGC (r = 0.64, *p* < 0.0001). Similar positive correlations were observed in residents’ procedural step-specific autonomy with LGS (r = 0.64, *p* < 0.0001) and LGC (r = 0.57, *p* < 0.0001).

All cases had completed matched records for OT, DC, and LOS. Seven out of 57 RAS cases did not have 30R data available at the time of data extraction. The mean of LOS was 6.49 h [95%CI 5.66, 7.28], OT was 80.46 min [95%CI 74.59, 86.32], and DC was $5,762.66 [95%CI 3531.62, 4203.74]. Likewise, eight cases (16.0%, 8/50) had 30R.

Overall, LGS had minimal impact on OT, LOS, and DC. However, notable overall differences in surgical outcomes were observed among three LGC scores (Table 2)—lower LGC scores associated with longer OT (*p* = 0.0221) and higher DC (*p* = 0.0231); a similar trend was also observed in LOS, though it was not statistically significant. Extreme sample comparison showed when compared to RAS cases with the highest LGC score (5—fully completed learning goal), cases with the lowest LGC score (3 – partially completed learning goal) had, on average, a 2.13-h longer LOS (8.00 > 5.87, *p* = 0.0496, power = 0.43), a 23.87-min longer OT (98.36 > 74.49, *p* = 0.0051, power = 0.80), and a $526.25 higher DC (4471.48 > 3945.23, *p* = 0.0064, power = 0.79). Interestingly, RAS cases with a score of 5 in LGC tend to have the highest probability of 30R—LGC 5: LGC 4: LGC 3 = 12.36%: 3.37%: 1.12%. The extreme comparison showed when particularly compared RAS cases with an LGC score of 5 to those with an LGC score of 3, cases with LGC score of 5 had a higher probability of 30R (18.03% > 1.64%), though this was not significant.

**Table 2:** Impact of learning goal completion (LGC) on RAS outcome measures

Learning goal completion score	Rating scale			*p* Value	Power (α = 0.05)
	3	4	5		
Length Of Stay (hours)	8.00	6.79	5.87	0.2052	0.33
Operative Time (mins)	98.36	81.46	74.49	0.0221	0.70
Direct Cost (dollars)	4471.84	4034.83	3945.23	0.0231	0.69

## Discussion

The use of robotic-assisted surgery (RAS) is rapidly increasing, simultaneously leading to a demand for enhancing resident robotic surgery training [17, 18] in order to secure the supply of high quality independent robotic surgeons for future patient care. To optimize the quality of resident training and the RAS care delivery, it is necessary to gain more knowledge about which intraoperative teaching/learning element(s) might influence robotic surgical care outcomes. Findings from this study suggest that the case-specific learning goal would not only influence resident intraoperative autonomy but also have an impact on LOS, OT, DC and 30R of outpatient RAS cases.

Setting an achievable case-specific learning goal that is appropriate for a given resident’s competency level and is mutually agreed upon by both the attending surgeon and the resident is crucial. This specific learning goal provides a clear roadmap toward the resident’s intraoperative learning target for a surgical case, helping to align the attending surgeon’s teaching with the resident’s learning efforts. If the attending surgeon agrees that the proposed learning goal is appropriate and manageable, they are typically willing to slow down and give the resident more autonomy to work on the learning goal or task, which is valuable for the resident’s competency development [19]. Therefore, further investigation into practice guidelines for RAS procedure-specific learning goals for residents becomes necessary.

Study findings also suggest that how well a resident completes their learning goal influences OT, DC, and LOS in the three RAS (RC, RIH, and RVH) study samples, which aligns with the literature [20–22]. If a resident cannot complete the learning goal or task selected for an RAS case, it may be because the attending surgeon or resident has inaccurately estimated the resident’s competency level and/or the case complexity, leading to the attending surgeon taking over the case. This is also supported by current data showing that LGC has a significant positive correlation with resident autonomy—“Overall Guidance” metric—(r = 0.64, *p* < 0.0001). Likewise, an ambitious learning goal commonly extends surgery duration, as the attending surgeon needs to slow down to teach and the resident also needs more time to safely practice skills, resulting in prolonged recovery time and increased cost [23].

Surprisingly, findings from this study suggest that RAS cases with a score of 5 in LGC tend to have the highest probability of 30R. One possible reason may be that a resident operating under high stress as the primary surgeon may make more mistakes in the OR according to the cognitive load theory [24–26]. Research shows that numerous medical errors affecting patient safety often result from complex human factors such as cognitive bias, time pressure, attention lapses, communication failure, insufficient skill, and fatigue [27–29]. Residents experience significant stress while operating as console surgeons under the supervision of an attending surgeon in RAS cases. According to the well-established Swiss Cheese Model of human error [30], an attending surgeon can help a resident fix all active errors—that is, errors made by the resident at the point of care—to ensure patient safety and operative efficiency in the OR. However, fixing all active errors may not prevent latent errors, which are literally "accidents waiting to happen" within 30 days of the operation [31]. Thus, current findings highlight the need to identify appropriate and achievable learning goals or tasks for residents based on their RAS experience, competency level, and cognitive load capacity to promote resident training and patient care simultaneously. It is also crucial to identify root causes of latent errors to optimize RAS outcomes without compromising resident intraoperative training [32].

This study has some limitations. First, it is a preliminary study with limited samples, leading to lack of strong overall statistical power. Second, we are unable to assess potential impact from patient-related factors on study findings. Third, it is a single institution study, and the local RAS patient population may influence the results. However, current study provides useful actionable information to help surgery residency programs optimize an intraoperative teaching protocol for RAS cases aiming to effectively teach a resident while maintaining the best RAS surgical outcomes. Lastly, we are unable to access and identify reasons for each patient’s 30R. Thus, we cannot exclude the possibility that the 30R is caused by other non-surgery related factors. Future study with a larger sample size is needed to further investigate the findings.

## Conclusion

Findings from this preliminary study suggest that the case-specific learning goal may influence resident autonomy as well as LOS, OT, DC and 30R of outpatient RAS cases (RC, RIH, and RVH). Identifying an achievable learning goal upon a resident’s competency level may enhance intraoperative training in RAS and surgical care outcomes.

## Data Availability

The data that support the findings of this study are available from the corresponding author upon reasonable request. Data is available from the corresponding authors with the permission of the medical center.

## References

[1] Reddington H, Bogursky A, Ballinger Z, Widdowson K, Guart J, Walter D, et al. Robotic surgery training during general surgery residency: a national survey study. J Surg Educ. 2025;82(11):103702. 10.1016/j.jsurg.2025.103702. 40961838 10.1016/j.jsurg.2025.103702

[2] Chen X, Wang TN, Sarin A, Patel A, Ali A, Samreen S, et al. Robotic vs. laparoscopic surgery at the operational level: an investigation of surgeons’ perspectives. Surg Endosc. 2025;39(11):7620–7. 10.1007/s00464-025-12152-y. 40897870 10.1007/s00464-025-12152-y PMC12618382

[3] Madion MP, Kastenmeier A, Goldblatt MI, Higgins RM. Robotic surgery training curricula: prevalence, perceptions, and educational experiences in general surgery residency programs. Surg Endosc. 2022;36(9):6638–46. 10.1007/s00464-021-08930-z. 35001224 10.1007/s00464-021-08930-z

[4] Porterfield JR Jr, Podolsky D, Ballecer C, Coker AM, Kudsi OY, Duffy AJ, et al. Structured resident training in robotic surgery: recommendations of the robotic surgery education working group. J Surg Educ. 2023. 10.1016/j.jsurg.2023.09.006. 37827925 10.1016/j.jsurg.2023.09.006

[5] Weigl M, Antoniadis S, Chiapponi C, Bruns C, Sevdalis N. The impact of intra-operative interruptions on surgeons’ perceived workload: an observational study in elective general and orthopedic surgery. Surg Endosc. 2015;29(1):145–53. 10.1007/s00464-014-3668-6. 24986016 10.1007/s00464-014-3668-6

[6] Antoniadis S, Passauer-Baierl S, Baschnegger H, Weigl M. Identification and interference of intraoperative distractions and interruptions in operating rooms. J Surg Res. 2014;188(1):21–9. 10.1016/j.jss.2013.12.002. 24405613 10.1016/j.jss.2013.12.002

[7] Kasotakis G, Lakha A, Sarkar B, Kunitake H, Kissane-Lee N, Dechert T, et al. Trainee participation is associated with adverse outcomes in emergency general surgery: an analysis of the National Surgical Quality Improvement Program database. Ann Surg. 2014;260(3):483–90. 10.1097/SLA.0000000000000889. (**discussion 490-3**). 25115424 10.1097/SLA.0000000000000889

[8] Krell RW, Birkmeyer NJ, Reames BN, Carlin AM, Birkmeyer JD, Finks JF. Michigan Bariatric Surgery Collaborative. Effects of resident involvement on complication rates after laparoscopic gastric bypass. J Am Coll Surg. 2014;218(2):253–60. 10.1016/j.jamcollsurg.2013.10.014. 24315885 10.1016/j.jamcollsurg.2013.10.014 PMC4004631

[9] Ferraris VA, Harris JW, Martin JT, Saha SP, Endean ED. Impact of residents on surgical outcomes in high-complexity procedures. J Am Coll Surg. 2016;222(4):545–55. 10.1016/j.jamcollsurg.2015.12.056. 26905188 10.1016/j.jamcollsurg.2015.12.056

[10] Benissan-Messan DZ, Tamer R, Pieper H, Meara M, Chen XP. What factors impact surgical operative time when teaching a resident in the operating room. Heliyon. 2023;9(6):e16554. 10.1016/j.heliyon.2023.e16554. (**May 21**). 37251464 10.1016/j.heliyon.2023.e16554 PMC10220402

[11] Roberts NK, et al. The briefing, intraoperative teaching, debriefing model for teaching in the operating room. J Am Coll Surg. 2009;208(2):299–303. 19228544 10.1016/j.jamcollsurg.2008.10.024

[12] Han AY, Naples R, French JC, Dragomirescu C, Tu C, Lipman JM. Operative teaching takes “GUTS”: impact of educational time out on trainee’s cognitive load. Am J Surg. 2022;224(3):851–5. 10.1016/j.amjsurg.2022.03.037. 35414429 10.1016/j.amjsurg.2022.03.037

[13] Childers CP, Maggard-Gibbons M. Understanding costs of care in the operating room. JAMA Surg. 2018;153(4):e176233. 10.1001/jamasurg.2017.6233. 29490366 10.1001/jamasurg.2017.6233 PMC5875376

[14] Intuitive Surgical Inc. Da Vinci Residency/Fellowship Training Certificate of Training Equivalency Requirements and Letter. Available from: residency-training-certificate-letter-210352.pdf. Accessed 23 Feb 2026.

[15] Chen XP, Harzman A, Cochran A, Ellison EC. Evaluation of an instrument to assess resident surgical entrustable professional activities (SEPAs). Am J Surg. 2020;220(1):4–7. 10.1016/j.amjsurg.2019.08.026. 31526512 10.1016/j.amjsurg.2019.08.026

[16] Chen XP, Cochran A, Harzman A, Ellison EC. Predicting prospective resident entrustment: from evaluation to action. Am J Surg. 2021;222(3):536–40. 10.1016/j.amjsurg.2021.01.020. 33485620 10.1016/j.amjsurg.2021.01.020

[17] Rodriguez PP, Kapadia S, Moazzez A, et al. Should robotic surgery training become a general surgery residency requirement? A national survey of program directors in surgery. J Surg Ed. 2022;79(6):e242–324. 10.1016/j.jsurg.2022.06.010 35831236

[18] Imai T, Amersi F, Tillou A, Chau V, Soukiasian H, Lin M. A multi-institutional needs assessment in the development of a robotic surgery curriculum: perceptions from resident and faculty surgeons. J Surg Educ. 2023;80(1):93–101. 10.1016/j.jsurg.2022.08.002. 36075804 10.1016/j.jsurg.2022.08.002

[19] Chen XP, Sullivan AM, Smink DS, Alseidi A, Bengtson JM, Kwakye G, et al. Resident autonomy in the operating room: how faculty assess real-time entrustability. Ann Surg. 2019;269(6):1080–6. 10.1097/SLA.0000000000002717. 31082905 10.1097/SLA.0000000000002717

[20] Sasor SE, Flores RL, Wooden WA, Tholpady S. The cost of intraoperative plastic surgery education. J Surg Educ. 2013;70(5):655–9. 10.1016/j.jsurg.2013.04.008. 24016378 10.1016/j.jsurg.2013.04.008

[21] Tschan F, Keller S, Semmer NK, Timm-Holzer E, Zimmermann J, Huber SA, et al. Effects of structured intraoperative briefings on patient outcomes: multicentre before-and-after study. Br J Surg. 2021;109(1):136–44. 10.1093/bjs/znab384. 34850862 10.1093/bjs/znab384 PMC10401893

[22] Meara M, Pieper H, Shields M, Woelfel I, Wang T, Renton D, et al. What influences general surgery residents’ prospective entrustment and operative time in robotic inguinal hernia repairs. Surg Endosc. 2023;37(10):7908–13. 10.1007/s00464-023-10242-3. 37430122 10.1007/s00464-023-10242-3

[23] Smith T, Evans J, Moriel K, Tihista M, Bacak C, Dunn J, et al. The cost of OR Time is $46.04 per minute. J Ortho Bus. 2022;2(4):10–3.

[24] Grantcharov PD, Boillat T, Elkabany S, Wac K, Rivas H. Acute mental stress and surgical performance. BJS Open. 2018;3(1):119–25. 10.1002/bjs5.104. 30734023 10.1002/bjs5.104 PMC6354185

[25] Crijns TJ, Kortlever JTP, Guitton TG, Ring D, Barron GC. Symptoms of burnout among surgeons are correlated with a higher incidence of perceived medical errors. HSS J. 2020;16(Suppl 2):305–10. 10.1007/s11420-019-09727-6. 33380961 10.1007/s11420-019-09727-6 PMC7749919

[26] Sergi CM. Medical errors can cost lives. Arch Med Sci. 2024;20(4):1378–83. 10.5114/aoms/192727. 39439691 10.5114/aoms/192727 PMC11493032

[27] Sameera V, Bindra A, Rath GP. Human errors and their prevention in healthcare. J Anaesthesiol Clin Pharmacol. 2021;37(3):328–35. 10.4103/joacp.JOACP_364_19. 34759539 10.4103/joacp.JOACP_364_19 PMC8562433

[28] Santos G, Jones MW. Prevention of Surgical Errors. 2023 May 29. In: StatPearls. Treasure Island (FL): StatPearls Publishing; 2025 Jan–. PMID: 37276278. 37276278

[29] Brennan PA, Oeppen RS. The role of human factors in improving patient safety. Trends Urol Mens Health. 2022;13:30–3. 10.1002/tre.858.

[30] Reason J. Human error: models and management. BMJ. 2000;320(7237):768–70. 10.1136/bmj.320.7237.768. 10720363 10.1136/bmj.320.7237.768 PMC1117770

[31] Wiegmann DA, Wood LJ, Cohen TN, Shappell SA. Understanding the “Swiss Cheese Model” and its application to patient safety. J Patient Saf. 2022;18(2):119–23. 10.1097/PTS.0000000000000810. 33852542 10.1097/PTS.0000000000000810 PMC8514562

[32] Woelfel I, Wang T, Pieper H, Meara M, Chen XP. Distortions in the balance between teaching and efficiency in the operating room. J Surg Res. 2023;283:110–7. 10.1016/j.jss.2022.10.032. 36402083 10.1016/j.jss.2022.10.032

